# Biosafety considerations and risk reduction strategy for a new veterinary faculty building and teaching hospital in Sweden

**DOI:** 10.1080/20008686.2020.1761588

**Published:** 2020-06-04

**Authors:** Martin Wierup, Ulrika Allard Bengtsson, Ivar Vågsholm

**Affiliations:** a Department of Biomedical Sciences and Veterinary Public Health, Swedish University of Agricultural Sciences; b Department of Microbiology, National Veterinary Institute (SVA)

**Keywords:** Biosafety, animal health, occupational health, veterinary teaching hospital design, regulatory framework

## Abstract

**Introduction:**

This paper describes a hazard- and risk-based strategy and recommendations on relevant biosafety levels in facility design of a new veterinary faculty building including a veterinary medical teaching hospital. Both animal and human health were considered.

**Materials and methods:**

Agents listed in the regulatory frameworks on animal and human health were identified as the main potential hazards. Suggestions on biosafety level and facility design were based on the official risk grouping of those agents, the associated risk management procedures, and biosafety experiences from previous faculty buildings.

**Results and Discussion:**

It was suggested that VHC should not be designed for work with agents requiring facilities at biosafety levels 3 and 4, and that actions in cases of accidental exposure to notifiable infections should follow the regulatory requirements. Facilities requiring biosafety level 2 were identified from risk scenarios and transmission routes.

Experiences from the first five years of operation revealed good prevention of spread of infection from patients in isolation facilities and successful elimination of Salmonella and MRSA from the large animal clinic.

**Conclusion:**

In order to avoid costly construction mistakes, an overall biosafety strategy should be formulated and used as guidance for architects and other relevant stakeholders designing facilities for the animal health sector. Regulatory requirements on infectious diseases must be complied with.

## Introduction

Research, teaching, and clinical training of students at a veterinary faculty frequently involve exposure to animal and zoonotic pathogens, and a risk for subsequent infections in both animals and humans. Numerous reports describe outbreaks of infections, particularly in clinical settings but also spread from laboratories, of agents like *Mycobacterium bovis* and Foot and Mouth Disease (FMD) [1–4]. Salmonella is a well-known cause of nosocomial infection [5,6] and, during recent years, a risk of spreading antibiotic-resistant pathogens has increasingly been reported [7–10]. Methicillin-resistant *Staphylococcus aureus* (MRSA) in equine hospitals is a concern for both animal and public health [11,12]. A veterinary faculty building and teaching hospital thus needs to be designed to allow for implementation of appropriate biosafety measures to protect both animals and humans.

Guidelines to avoid nosocomial infections in human hospital and laboratory facilities are known [13–16], and can also be applied in the veterinary sector, in particular for the prevention of occupational infections. Biosafety reports or guidelines for animal hospitals have been published [17–23]. However, none of these covers the design of veterinary faculty buildings, which in addition to a teaching hospital with ambulatory practice also include facilities for post mortem examination, microbiology, anatomy, and obstetrics. In addition, to our knowledge there are no corresponding guidelines or reports that consider the regulatory requirements and responsibilities for actions in cases of accidental exposure or outbreaks of diseases classified as dangerous to animals and public health.

This paper presents a novel hazard- and risk-based approach developed for identification of relevant biosafety levels of a planned veterinary faculty building combined with an animal teaching hospital. The approach was based on assessment of the regulatory requirements for prevention and control of infectious diseases in both animals and humans. Some biosafety risk functions were highlighted and some approaches for relevant risk-reducing design and management were suggested. These suggestions were based on experiences from previous veterinary faculty buildings, obtained through interviews with individuals responsible for those functions. The outcome of the biosafety plan following five years of implementation was also assessed through repeat interviews with those key individuals and with key managers.

## Material and methods

### Buildings and facilities

**Figure 1.**
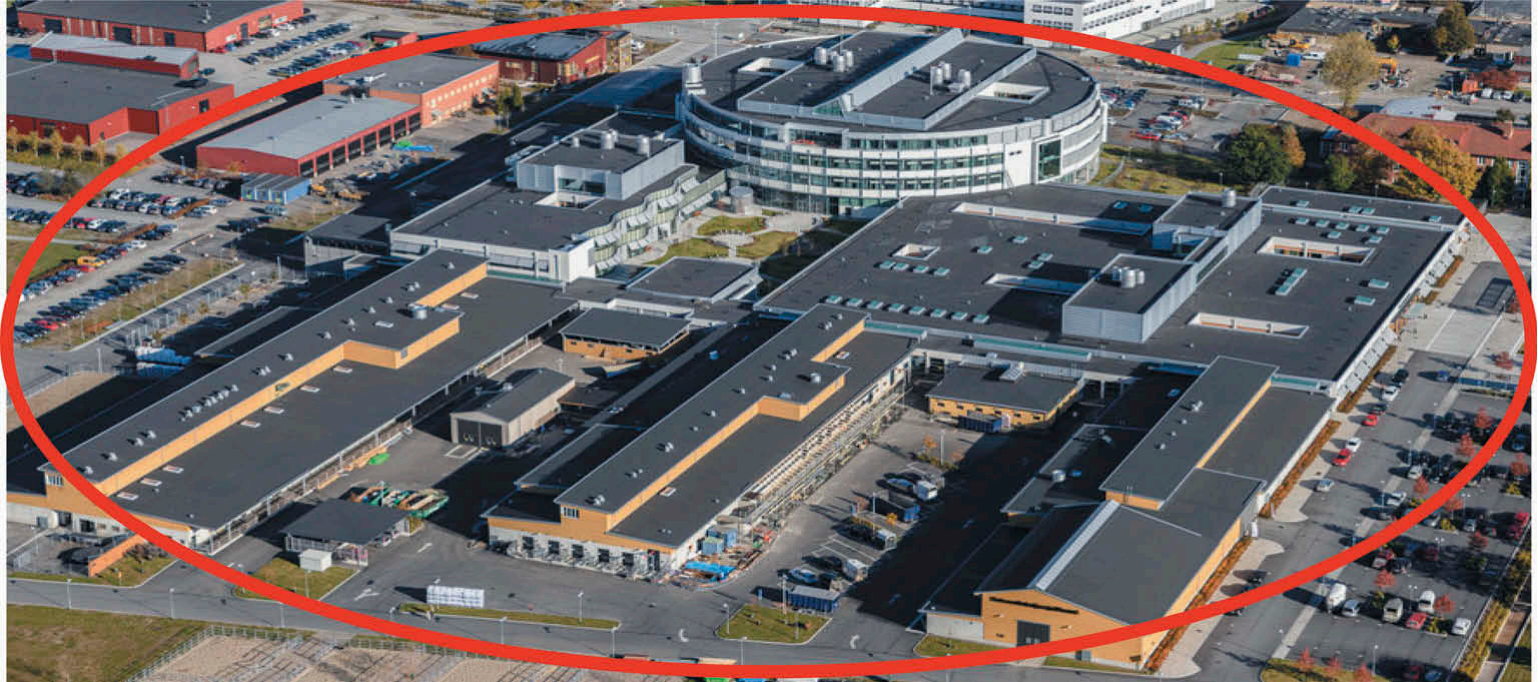
Aerial view of the centre for Veterinary Medicine and Animal Science (VHC), Swedish University of Agricultural Sciences, Uppsala, Sweden. For further information, see Figure 2. (Source ISBN 978-91-576-9344-0).

**Figure 2.**
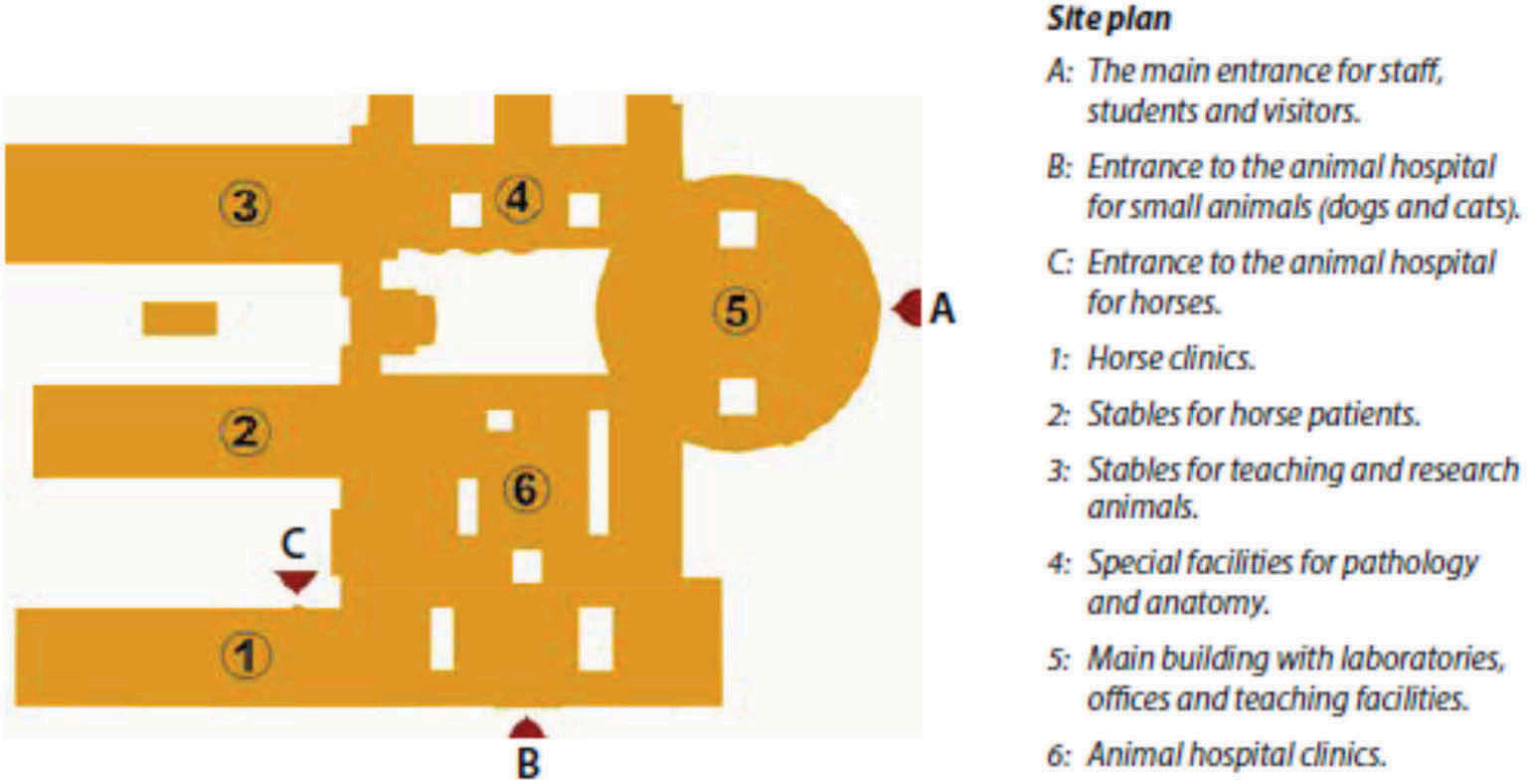
Site plan for the centre for Veterinary Medicine and Animal Science (VHC). For further information, see Figure 3. (Source ISBN 978-91-576-9344-0).

The subject of the assessment was all the buildings and associated facilities for a new veterinary faculty building (Centre for Veterinary Medicine and Animal Science; hereafter referred to as VHC) at the Swedish University for Agricultural Sciences (SLU), Uppsala, Sweden. In addition to a university teaching hospital for small and large animals and a large animal ambulatory practice (here referred to as VMTH), VHC had to include conventional office spaces, lecture halls, research laboratories for different faculty departments, and special facilities for post mortem examinations, microbiology, anatomy, and obstetrics, with supporting infrastructure. VHC was planned to provide for the education of some 1,000 students in veterinary medicine, animal science, and veterinary nursing programs. The completed VHC complex has a total area of 53 000 m^2,^ distributed over six connected sections, of which one is VMTH (Figure 1–3). The main architectural design was decided in 2008, followed by detailed planning of the functions, space, and when relevant, biosafety required by each department.

**Figure 3.**
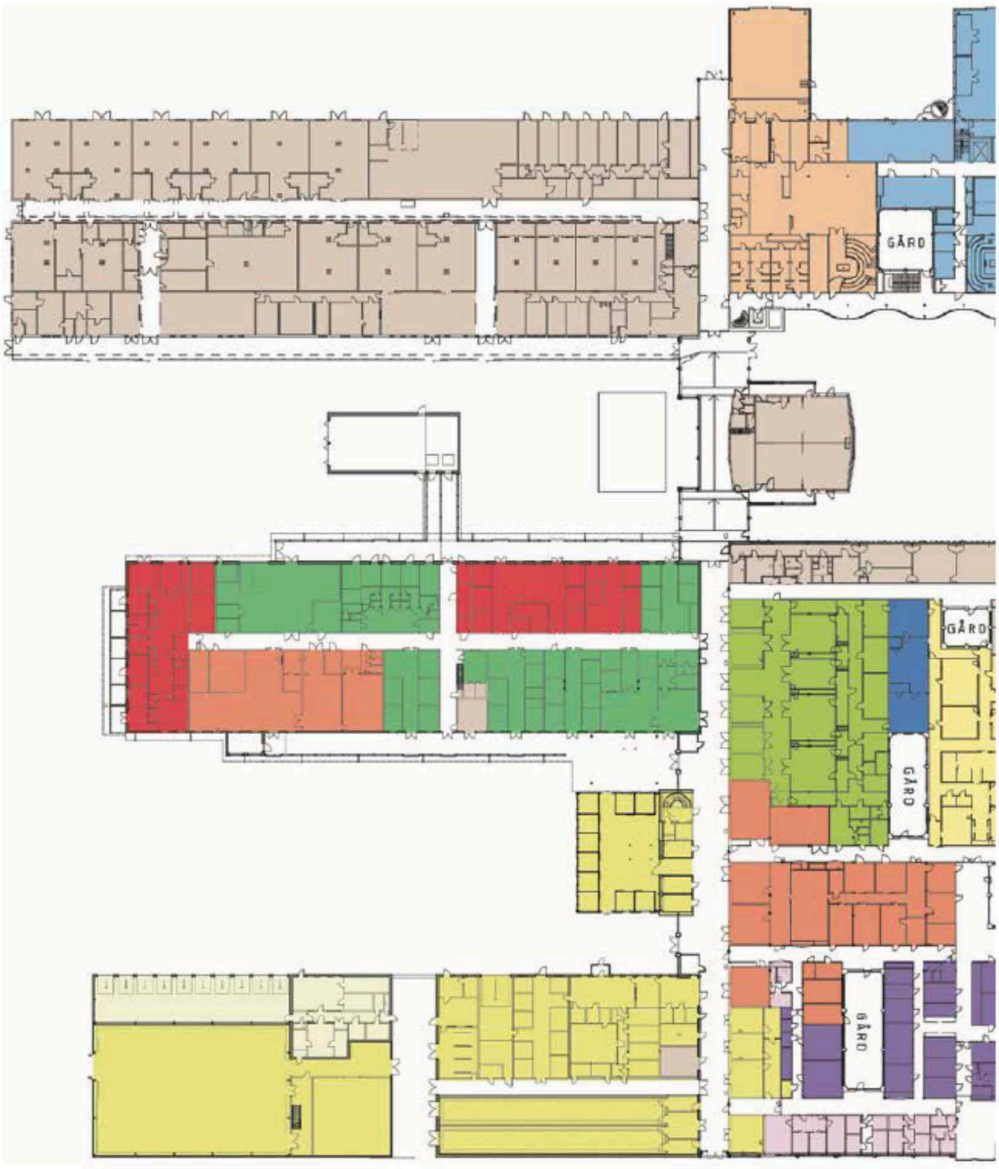
Detailed site plan for the centre for Veterinary Medicine and Animal Science (VHC). The diagram continues below. (Source ISBN 978-91-576-9344-0).

**Figure 3.**
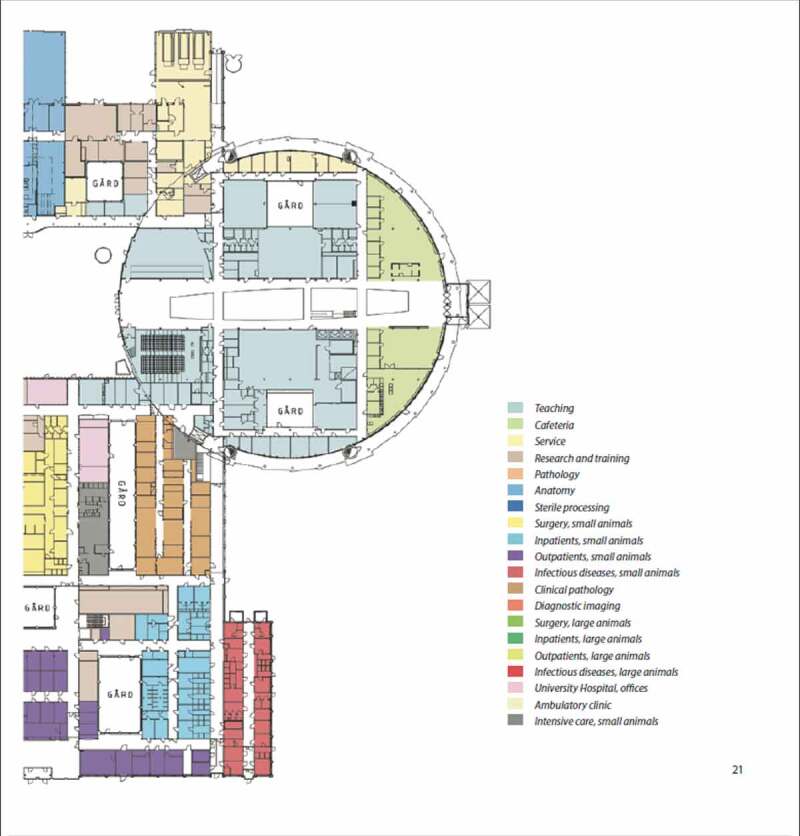
Continued.

The starting point for the present analysis was a biosafety strategy plan for VHC developed in 2009 [24]. The plan, which involved all relevant stakeholders including the academic staff, suggested modifications to the pre-final design that were considered in the final design of the buildings. VHC opened in 2014 and has been fully operating since then, allowing for assessment of the first five years of experiences of implementation of the biosafety plan.

Figure 1–3. Images of the centre for veterinary medicine and animal science (VHC). [Separate files].

### Hazard identification

**Figure 4.**
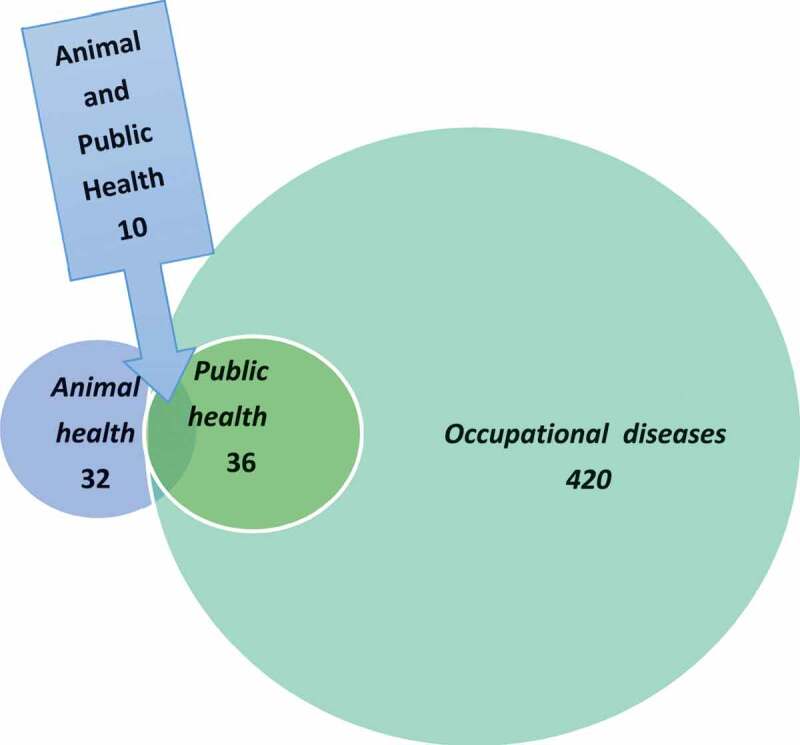
Number of diseases listed by relevant national agencies in Sweden in 2019 as dangerous to animal health, dangerous to public health and society, and occupational diseases. For further information, see Table 1.

The hazards considered in the biosafety plan concerned exposure of animals and humans to biological agents (bacteria, viruses, prions, parasites, and fungi) that can cause diseases in animals and to agents that can cause disease in, and be transmitted between, animals and humans, i.e. zoonoses. These notifiable diseases are categorized as occupational hazards, communicable human diseases, or animal pathogens. The numbers and interrelations of these three groups of diseases are illustrated in Figure 4 and Table 1. Agents only pathogenic to humans were excluded from the biosafety plan.

**Table 1.** Number of diseases in 2019 listed by relevant national agencies in Sweden as significant for animals and humans. Some agents are listed as significant for both animals and humans, but given different risk ratings.

		Listed/notifiable animal diseases (Swedish Board of Agriculture)			
				Dangerous to animal health Act on Epizootic Diseases	Dangerous to animal health Act on zoonosis
Animal health		Total 188		31	1
Human and Public Health	Listed occupational diseases (Risk groups 2–4)	Listed/notifiable communicable diseases (Public Health Agency of Sweden)			
	(Swedish Work Environment Authority)	Total	Mandatory contact tracing	Dangerous to public health/Risk group 3	Dangerous to society/Risk group 4
	420	67	50	33	3

#### Animal health

##### Listed diseases

In Sweden, there is no risk-based classification of all potential animal pathogens as applied for human pathogens (see below). Instead, any incidence of agents and diseases found to be of special importance primarily to food-producing animals or agents with zoonotic potential must be reported to the animal health authority (Swedish Board of Agriculture [25]). Based on factors like contagiousness and economic or zoonotic importance, some of these notifiable diseases are further prioritized and listed by legislation on epizootics [26] and zoonoses [27] (Table 1). Some of the diseases are also prioritized by regulations prescribing often very detailed and rigorous control measures, as a rule aiming at rapid eradication e.g. in the case of outbreaks of highly transmissible epizootic diseases like FMD, African swine fever, and highly pathogenic avian influenza. For European Union (EU)-harmonized diseases, control is in accordance with acting EU directives and, when applicable, World Organization for Animal Health/OIE recommendations.

##### Non-listed diseases

Non-listed pathogens or diseases cover a wide range of infections and include e.g. endemically occurring respiratory and gastrointestinal infections.

#### Public health – occupational infections

##### Risk classification

Agents considered to be potential human pathogens are listed in the Swedish Work Environment Act [13], which is based on an EU directive [16]. Both regulations also include risks related to exposure to agents in the animal healthcare sector. The agents are classified into four risk groups, although the classification of zoonotic agents only takes into account disease in humans (Table 1):

Risk group 1 agents are unlikely to cause disease in humans.

Risk group 2 agents can cause disease in humans and may pose a hazard to workers, but spread to the community it is unlikely. This risk group includes most of the pathogenic viruses, bacteria, and fungi, and includes e.g. herpes simplex, MRSA and non-typhoid Salmonella (in total 301 agents).

Risk group 3 agents can cause severe human disease, present a serious hazard to workers and may also spread to the community. This group includes agents causing e.g. anthrax, brucellosis, ornithosis, tularemia type A, EHEC, tuberculosis, typhoid fever, dysentery, rabies, and bovine spongiform encephalopathy (BSE) (in total 100 agents).

Risk group 4 agents are a serious hazard to workers and may present a high risk of spread to the community. This group includes e.g. smallpox, Lassa fever, ebola virus and other viral hemorrhagic fevers (in total 12 agents).

In total, approximately 420 pathogens are listed as potential occupational hazards, including 160 bacteria, 150 viruses, 6 prions, 70 parasites, and 30 fungi.

#### Public health – communicable diseases

For the Swedish public health situation, a limited number of risk group 3 and 4 diseases from the Work Environment Act [13] are listed as notifiable according to the Communicable Diseases Act and Communicable Diseases Ordinance [33]. Some of these notifiable diseases are further prioritized into three different categories, mandatory contact tracing (50), dangerous to public health (28) and dangerous to society [3], as summarized in Table 1.

#### Public health – zoonoses

Thirty (83.3%) of the diseases listed as dangerous to public health or to society and 11 (33.3%) of the diseases listed as dangerous infections in animals are zoonoses [26,27] (Table 1). However, only 10 (33.3%) of the zoonoses listed as dangerous to public health or to society are also listed as dangerous infections in animals (Figure 4). Agents that are pathogenic to both humans and animals may thus have different prioritizations in the animal and human health perspectives. For example, brucellosis is given a high priority both as a human pathogen (risk group 3) and as an animal pathogen (listed in the Act on Epizootic Diseases). The situation differs e.g. for Salmonella, which only is listed as notifiable as a human pathogen, but is given high priority as an animal pathogen (listed in the Act on Epizootic Diseases). This reflects the fact that in Sweden, a non-acceptance policy is applied for salmonella infections in food-producing animals. The opposite applies for diseases like ornithosis, tularemia type A, and EHEC, which are all given a higher priority (risk group 3) as human pathogens than as animal pathogens. These differences mainly reflect the lack of effective control measures for those agents in different animal populations or the environment, in contrast to the situation for Salmonella.

## Occurrence

Sweden is declared free from most of the diseases included in the Act on Epizootic Diseases, which thus do not normally occur in Sweden, while several of the remaining listed diseases may or do occur [29]. Salmonella, which is currently the only pathogen included in the Act on Zoonosis [27], rarely occurs in farm animal species in Sweden [30]. Remaining non-listed pathogens include a wide range of agents, which often show endemic occurrence. The listed human pathogens (risk groups 3 and 4) occur rarely in Sweden. The agents in risk group 2 may normally occur in both healthy and diseased human and animals.

## Biosafety levels – regulatory requirements

Identification of a relevant biosafety level for VHC was based on its specifications and had to distinguish between exposure following planned work with specific agents, e.g. in a laboratory, and exposure e.g. to potentially infected animals attending VMTH. The regulatory requirements on listed infectious diseases also needed to be complied with.

### Biosafety requirements for animal and human pathogens

Guidelines to ensure occupational biosafety in laboratory settings as regards risk group 2 agents must correspond to the standards for conventional microbiological laboratories, while risk groups 3 and 4 require additional biosafety, in biosafety level (BSL) 3 and (BSL4) laboratories, respectively [13]. In the Nordic countries, there is only one BSL4 laboratory [31] Work with agents causing the most contagious animal diseases, which normally only infect animals, require special high-containment and internationally approved laboratories [32,28]. In Sweden, high-containment laboratories for the veterinary sector are only available at the National Veterinary Institute (SVA), which can also handle all listed serious animal diseases [32] (Table 1) and initial work with samples from suspected FMD outbreaks. A regulatory framework also applies for clinical and other non-laboratory settings, including the facilities at VHC [13]. Additional regulations apply for the prevention of infections in humans [33]. Due to the lack of official guidelines for the animal sector, when devising a risk reduction strategy for VHC, guidelines from a One Health perspective were considered to be applicable also in prevention of transmission of animal infections.

## Risk scenarios and transmission routes

### Transmission routes into VHC

The major routes of introduction of pathogenic agents were identified as being through infected animals admitted as patients or for teaching or research, and by contaminated dead animals or animal organs for teaching, post mortem examination, or research. Contaminated feed was also considered a potential source of introduction, as were healthy human carriers, including staff, students, animal owners, and other visitors. Individuals with close contact with animals outside VHC, in Sweden or abroad, and teachers and students returning from farm visits as part of their clinical training were considered a special risk group.

The probability of exposure was assumed to be reflected by the animal and public health situation in Sweden. However, agents not normally occurring in Sweden may enter due to international travel or imports of animal products, food, and feed ingredients. Specimens for research, in particular from developing countries, were considered a potential high-risk source for the introduction of exotic agents. Moreover, it was assumed that infections by agents normally endemic in Sweden, like strangles in horses, might be introduced if animals in a fulminant stage of an infection were allowed to enter VHC without any risk-reducing precautions.

### Routes within VHC

It was assumed that an agent introduced to VHC could be further transmitted, by direct or indirect contact, within the facilities, e.g. by movement of live or dead animals, individuals, manure, feed, ventilation, and equipment. The possible spread of food-borne pathogens to and from catering facilities within VHC received special attention.

### Routes from VHC

A major potential route of spread from VHC was considered to be via different forms of waste (manure, urine, sewage, water, and laboratory waste). Other routes considered were infected live animals from the clinics or contaminated dead or euthanized animals leaving VHC for post mortem examination at the nearby SVA facilities (see below). Accidental release of agents from laboratories would be a potential and serious risk if recommended biosafety measures were not in place.

### Interviews

Experiences from the previous faculty building were gathered through personal interviews in 2009 with key individuals responsible for the facilities and functions requiring BSL 2 (see below) and on-site demonstrations of the biosafety routines applied. These key individuals were re-interviewed in 2019, when key individuals responsible for the overall management of VHC were also interviewed, to follow up on the first five years of biosafety experiences in VHC.

## Results

### Conclusions on appropriate biosafety level for VHC

The planned use of VHC did not include clinical or laboratory diagnostic work with animal or human pathogens requiring BSL 3 or 4 facilities, as SVA is tasked with that kind of diagnostic work in Sweden. Thus VHC was planned for work only with agents requiring facilities BSL 1 and 2. Due to the lack of risk classification of agents only infecting animals, a risk assessment by appropriate methods was prescribed to ensure that the recommended biosafety can be achieved when working with such agents at VHC [13]. In doubtful cases and for unknown agents, e.g. currently non-listed viruses, it was recommended that advice be sought from the relevant authorities.

The management of suspected cases of any notifiable animal infections requiring biosafety levels 3 or 4 must be based on the regulatory requirements, including reporting to the competent Swedish authority [25]and referral to the appropriate facility at SVA. The veterinarian must also immediately report suspicions of exposure to human pathogens in risk groups 3 or 4 to the regional medical officer (13; §17). When following these procedures, the responsibility for subsequent risk management is transferred to the competent authority for animal and public health, respectively.

## Facility design

In the proposed strategy for VHC, the following facilities and associated functions were suggested to require biosafety level 2 : (1) VMTH, (2) anatomy, obstetrics, and gynecology facilities managing carcasses and organs; (3) post mortem examination facilities and associated laboratories, (4) microbiological laboratories for research and teaching, and (6) houses and other facilities for mammals and aquatic animals [13]. It was recommended that the design of these facilities be based on available guidelines on biosafety (see above), or other recommendations given in the biosecurity report [24]. Some of the risk-reducing measures suggested for VMTH and some additional transverse functions not covered in available guidelines are described below.

## Biosafety of the VMTH

### Access limited to authorized persons

It was recommended that access to biosafety level 2 facilities be limited to authorized persons and include barrier precautions, with a clear separation between outside/clean and inside/dirty zone and change to suitable protective clothes and footwear. Thus, all students and staff entering these facilities, including VMTH, should only enter and exit at a designated point following a set protocol. The entry and exit point had to be welcoming, spacious, adapted to the number of staff and students, and well ventilated. In particular, the design had to allow for a natural logistic barrier between clean and dirty areas, and associated hygiene procedures.

### Carcasses and organs for teaching and post mortem examination

Special attention was recommended for the transport of carcasses and organs used for teaching in anatomy, obstetrics, and gynecology. Apart from safe sourcing of healthy animals, hygienic protocols and routines for their intake to VHC, possible section of carcasses or organs, storage, and exit from VHC for incineration were recommended in the risk reduction strategy. A special entrance room with elevator and cleaning facilities for transporting trucks was considered essential. Within VHC, it was suggested that organs be transported and stored in closed containers, and that a dishwasher be installed for cleaning, including possibly outgoing containers for transport. In a corresponding way, biosafety transport protocols had to be worked out for transport of dead animals from VMTH to post mortem examination or incineration.

### Suspected patients rejected or in isolation stables

Isolation protocols have been shown to significantly reduce the incidence of hospital-acquired infections [17]. Thus according to risk reduction strategy for VHC, animals with clinical signs or epidemiological links to outbreaks of transmissible infections should not be allowed to enter the main facilities. For such risk patients, separately located isolation houses were recommended, with direct access from outside and with barrier precautions, including stringent hygiene management protocols [22]. Ensuring the clinical skill for identifying such patients was highlighted. Contingency plans on how to manage such patients, should they be identified after entry to the clinics, were also suggested.

### Returning from farm and field visits

Veterinarians, students, and others returning to VHC and in particular to VMTH from farm visits present a risk of introducing infections to VMTH and, moreover, to other farms on subsequent visits. Relevant protocols and associated facility design to ensure the biosafety for this function were needed [22]. The risk reduction strategy recommended that returns should only be allowed at designated places with a ‘dirty’ area, where veterinarians and students would wash and change clothes before entering the ‘clean’ or interior side of the clinic. Well-planned design of both sides was advised, to ensure a simple procedure for washing of footwear and clothes and corresponding routines for equipment, samples, and accessories, and safe disinfection routines before reuse. Regular cleaning and disinfection of the medical equipment, as specified in a biosafety plan, was recommended [22].

## Manure management

Manure removal requires special attention in large animal facilities, due to its volume and the risk of contaminating the environment. The risk reduction strategy suggested that the manure be hygienically stored in containers under roof in a locked building well separated from the animal house. It also suggested that culverts for mechanical transport of manure should be accessible over their full length, to allow for cleaning and disinfection in cases of e.g. salmonella infections [5,6]. Cleaning and disinfection of culverts on a regular basis were recommended, to avoid the build-up of spots of manure and persistent microbial contamination. Using an increase in environmental contamination with Salmonella as an early warning of problems with the hygiene, as suggested by [21], was therefore not deemed applicable for VMTH.

## Wastewater management

Drains are a site for bacterial colonization and should therefore be routinely disinfected [18]. The drains at VHC were planned to be connected to the municipal sewage system and water treatment plants. This is a safeguard, but outbreaks of FMD virus from a laboratory have occurred through an inadequate drainage network [3,4].

Due to the suggested biosafety level for VHC and the dilution effect of municipal sewage system, in the risk reduction strategy it was deemed unnecessary to install facilities for preparedness for disinfection of wastewater, which has been proposed in guidelines for certain facilities in human hospitals [34]. The relevance of these guidelines has been questioned, because the combined pathogen load to the local municipal sewage system from all households and activities outside human hospitals in a city is often greater than the contribution from a single hospital [34].

A corresponding situation was assumed to exist for the release of animal pathogens from VMTH and the post mortem examination facilities at VHC, and reasonably also when considering microbe release from animal production farms.

## Hygiene

Regular professional cleaning was prescribed to minimize microbial contamination and colonization of the VHC environment and prevent the build-up of contaminated spots [18]. Transmission of infections agents via contact is one of the greatest biosafety concerns, particularly at public and animal health clinics [18,35]. Numerous studies have also verified the importance of hand washing. The single most efficient method to limit the spread of nosocomial infections in human healthcare by is hand washing and/or disinfection before and after treating each patient. Therefore the risk reduction strategy for VHC recommended that special attention be paid to providing convenient hand-washing stations, with antiseptic and soap dispensers at all significant locations within VHC. From a food safety perspective, it was also considered important to highlight the hygiene routines and washing stations at all the different catering facilities within VMTH and at different places in VHC. The strategy stated that, as standard, only foot-pedal or infrared sensor taps should be installed, to allow for hands-free access to tap water.

### Follow-up – five years of experience

Overall, the VHC largely functioned as planned. For the biosafety areas highlighted in the risk reduction strategy, the following experiences and discrepancies were observed:

- Isolation facilities. The small animal isolation unit was frequently fully occupied during the winter season by Salmonella-infected cats and the large animal isolation unit had to be extended by five additional boxes. No spillover of infections was observed between isolated animals and animals in the units for ordinary patients. In the large animal facilities for ordinary patients, Salmonella and MRSA infection in horses were successfully controlled [36]. For EU accreditation of the veterinary education, an extra protective fence had to be installed to prevent contact with possibly rabies-infected small animals if allowed to enter the outdoor fenced area [37].
- Ventilation. No air-cooling system was installed in the large animal houses, which led to major health problems during the warm season when doors and windows were left open to reduce the temperature. This resulted in malfunction of the ventilation, spread of contaminated air between units, and subsequent problems with viral respiratory infections. Financial compensation had to be paid to horse owners, and additional costs are foreseen for installation of an air-conditioning system.
- Effluent water. Although not specifically recommended [24], tanks for sterilization of effluent water were installed in both the small animal clinic and the post mortem examination rooms. In the small animal clinic, the sterilization facilities were never used and in the post mortem examination facilities they are considered to be both costly and unnecessary.
- Conflicting requirements. During the planning process, formal requirements on emergency exits were in conflict with the hygiene barriers in the theatre for demonstration of carcasses in the pathology and anatomy departments. However, the planned biosafety design of barriers was maintained and possible emergency situations are resolved on a case-by-case basis.
- Hands-free taps. Despite the strong recommendation to only install hands-free taps in VHC, ordinary manual taps were also installed, e.g. in the large animal clinic, including in the isolation stables for infected horses and in fact also later in the expanded stables.

## Discussion

This paper describes relevant biosafety levels suggested for a new veterinary faculty building (VHC) combined with a university teaching hospital (VMTH) in Sweden. Relevant risk-reducing designs and management options suggested for biosafety risk functions of special importance are also described. Formulation of the biosafety plan required consideration of the regulatory frameworks on both animal and human health, thus differing from strategies for corresponding institutions in the public health sector. The management of possible exposure to highly infectious animal diseases (e.g. FMD, African swine fever and highly pathogenic avian influenza (Table 1) was highlighted as requiring special attention. Another area identified as requiring special attention was exposure to zoonotic agents, for which the regulatory risk classifications and associated management frequently differ between animal or public health perspectives. In order to avoid suboptimal design and costly construction mistakes, a hazard- and risk based approach was applied to assess the planned operations at the VHC facility.

A limiting factor for risk characterization of disease-causing agents was the lack of official guidance in Sweden on prevention and control of all potential animal pathogens, which is available e.g. in the UK [38]. Applying a One Health perspective, the guidelines for protection of workers [13] were therefore suggested as suitable also for the prevention of infections in animals.

Because the planned use of VHC did not include clinical or laboratory diagnostic work with pathogens dangerous to animal or human health, it was recommended that VHC should not be planned or designed for work with agents requiring biosafety levels 3 and 4. Moreover, the risk reduction strategy suggested no specific facility design or equipment at VHC for the possible management of agents above risk group 2 or for diseases listed as dangerous to animal health (Table 1). This recommendation was based on the statutory requirements regarding the management of suspected cases of any notifiable animal and human infections, which include reporting to the competent Swedish authorities [13,25].

Experiences from the first five years of full operation of VHC showed that the facility design largely followed the suggestions made in the risk reduction strategy [24]. Deviations found were probably due to tradition and budgetary concerns. Surprisingly, manual taps were installed at several locations, including in the isolation stables for infected horses. Tanks for sterilization of effluent water (not recommended by the strategy) were installed, but later found to be both costly and unnecessary [34]. A valuable but costly lesson was that lack of air-cooling in the large animal houses resulted in the spread of respiratory viral infections in hot weather. Costly construction mistakes were thus not fully avoided.

However, the suggested biosafety level was fully adopted. The recommended facilities and biosafety management for isolation of infectious patients upon admittance to VMTH have so far prevented spread of infections to other hospital facilities. Unforeseen outbreaks of Salmonella and MRSA from the large animal clinic have also been successfully controlled [36].

## Conclusions

Planning, building, and operation of veterinary college buildings that including teaching hospitals is a complex process. In order to avoid costly construction mistakes, a biosafety strategy should be formulated and considered from the start of the design stage and during the whole construction process. In the absence of such a strategy document, there is a risk of the design being guided by opinions from individual faculty members and other stakeholders, which may result in suboptimal design due to disproportionate estimates of the requirements for management of potential microbiological risks. As novel pathogens to humans and animals are continually emerging, the biosafety risks should be re-assessed at regular intervals.
